# Supplementary figures and images for: The polyol pathway is an evolutionarily conserved system for sensing glucose uptake
Source: PLoS Biol. 2022 Jun 10;20(6):e3001678. doi: 10.1371/journal.pbio.3001678 (PMC9223304; doi:10.1371/journal.pbio.3001678)

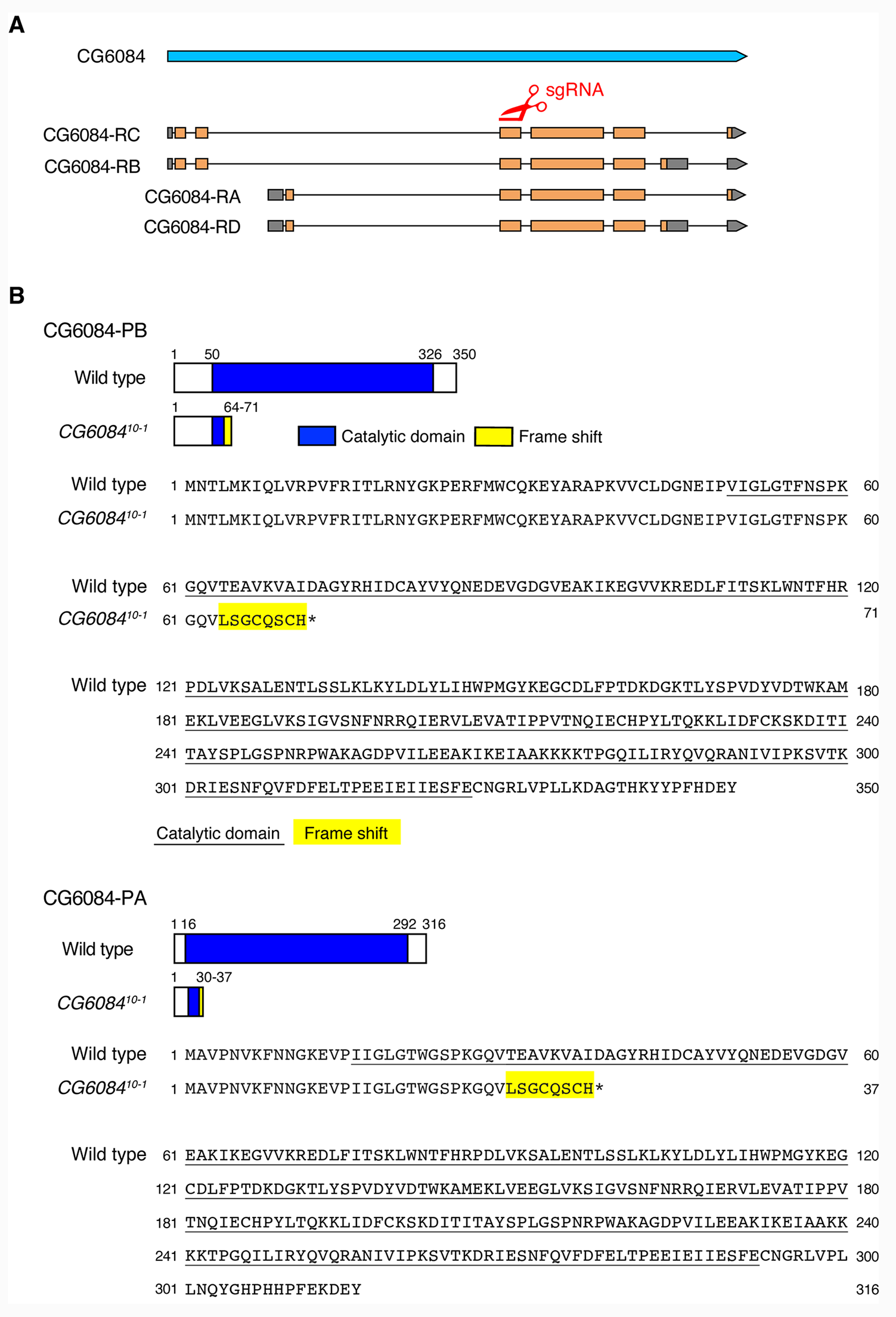

Supplement: S1 Fig — (A) CRISPR-mediated mutagenesis of the CG6084 gene. A sgRNA was designed for the sequence within the exon common to the CG6084 isoforms. The genomic map was adapted from FlyBase (http://flybase.org). (B) Breakpoint of the CG608410-1 allele. The CG608410-1 mutation caused a frameshift (yellow) leading to a premature termination in all isoforms of the CG6084 protein. The mutant proteins lack most of the catalytic domain of the CG6084 protein (blue in schematic, underlined in the amino acid sequence). (TIF) [file pbio.3001678.s001.tif]

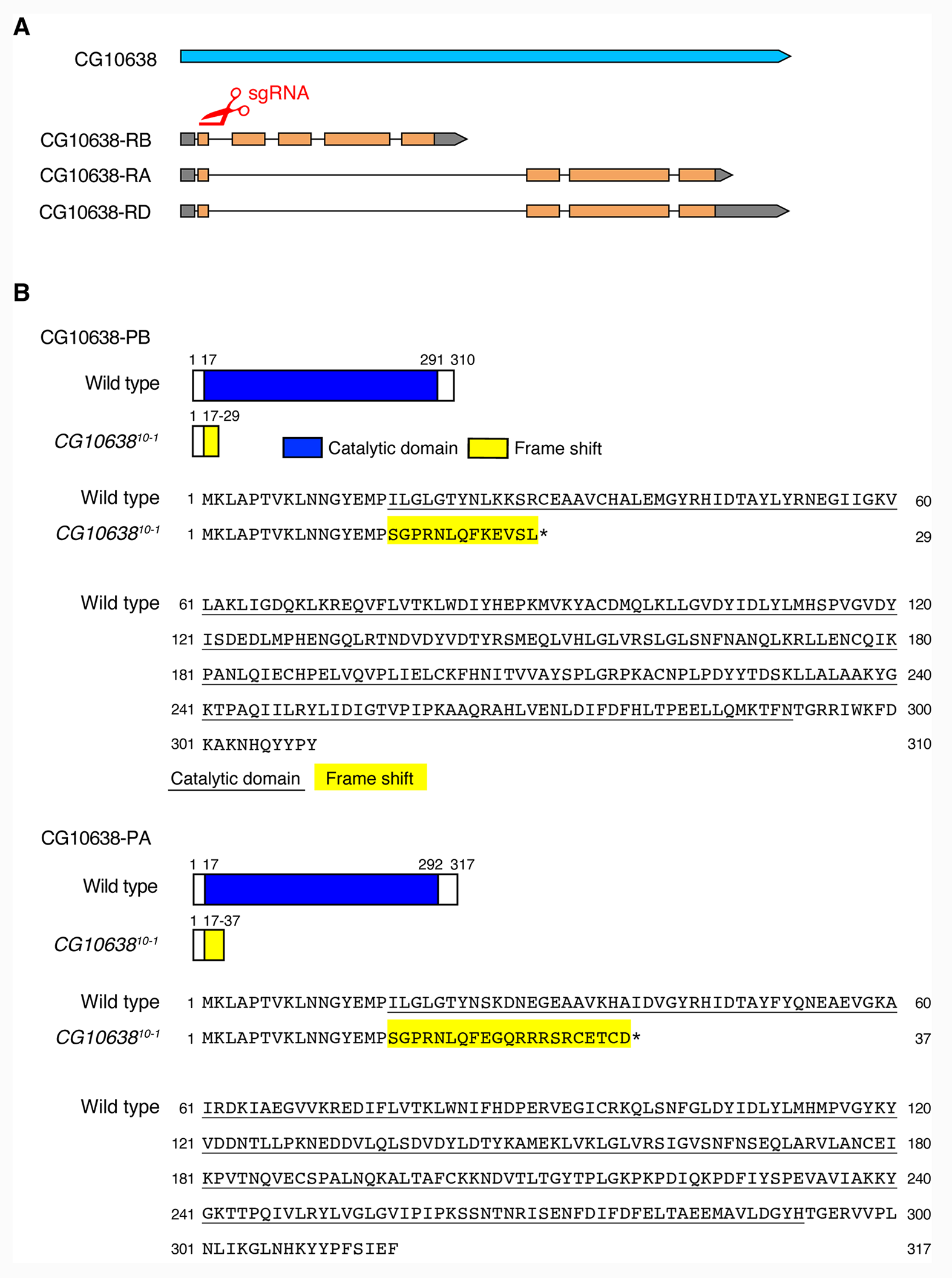

Supplement: S2 Fig — (A) CRISPR-mediated mutagenesis of the CG10638 gene. A sgRNA was designed for the sequence within the exon common to the CG10638 isoforms. The genomic map was adapted from FlyBase (http://flybase.org). (B) Breakpoint of the CG1063810-1 allele. The CG1063810-1 mutation caused a frameshift (yellow) leading to premature termination of all isoforms of the CG10638 protein. The mutant proteins lack most of the catalytic domain (blue in schematic, underlined in the amino acid sequence). (TIF) [file pbio.3001678.s002.tif]

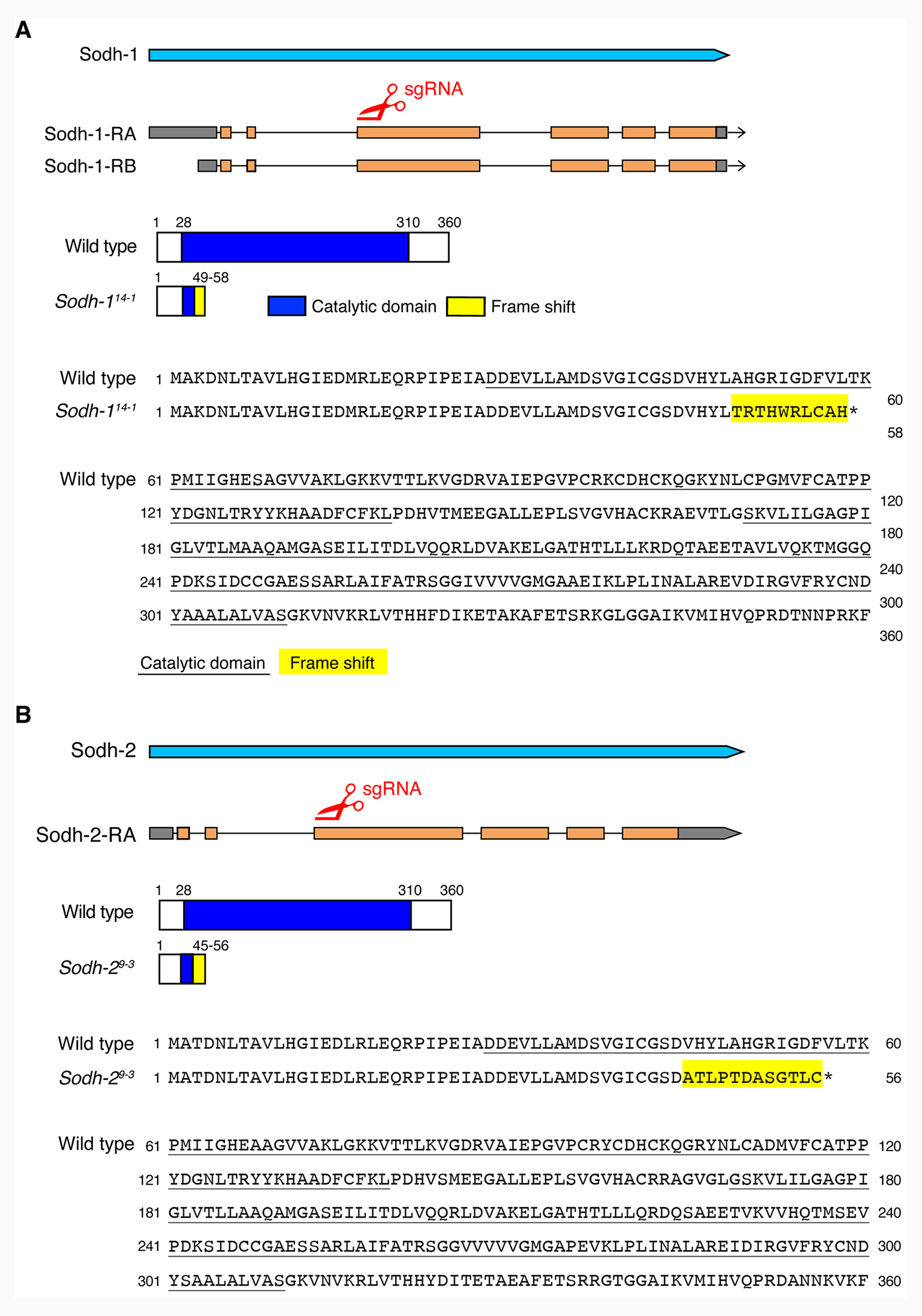

Supplement: S3 Fig — (A) CRISPR-mediated mutagenesis of the Sodh-1 gene. A sgRNA was designed for the sequence within the exon common to both isoforms of Sodh-1. The Sodh-114−1 mutation caused a frameshift (yellow) leading to premature termination of both isoforms of the Sodh-1 protein. The mutant proteins lack most of the catalytic domain (blue in schematic, underlined in the amino acid sequence). (B) CRISPR-mediated mutagenesis of the Sodh-2 gene. A sgRNA was designed for the sequence in the third exon of Sodh-2. The Sodh-29−3 mutation caused a frameshift (yellow) leading to premature termination of the Sodh-2 protein. The mutant proteins lack most of the catalytic domain (blue in schematic, underlined in the amino acid sequence). The genomic maps in (A) and (B) were adapted from FlyBase (http://flybase.org). (TIF) [file pbio.3001678.s003.tif]

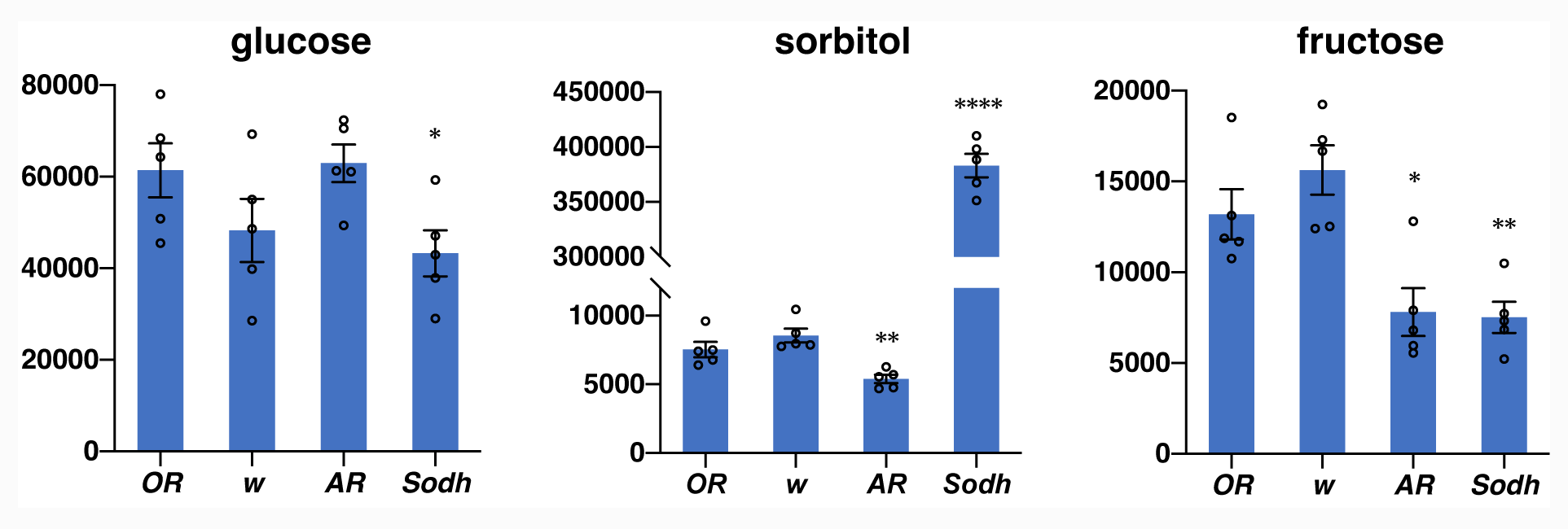

Supplement: S4 Fig — The amount of glucose, sorbitol, and fructose contained in the hemolymph of AR and Sodh mutant third instar larvae was measured using GC/MS. OR and w were used as controls; 10 larvae per batch, n = 5 batches for all experiments. Bar graphs show mean ± SE. The results of statistical significance tests between OR and AR or Sodh mutants are shown. *P < 0.05; **P < 0.01; ****P < 0.0001. The data underlying the graphs can be found in S1 Data. AR, aldose reductase; Sodh, sorbitol dehydrogenase. (TIF) [file pbio.3001678.s004.tif]

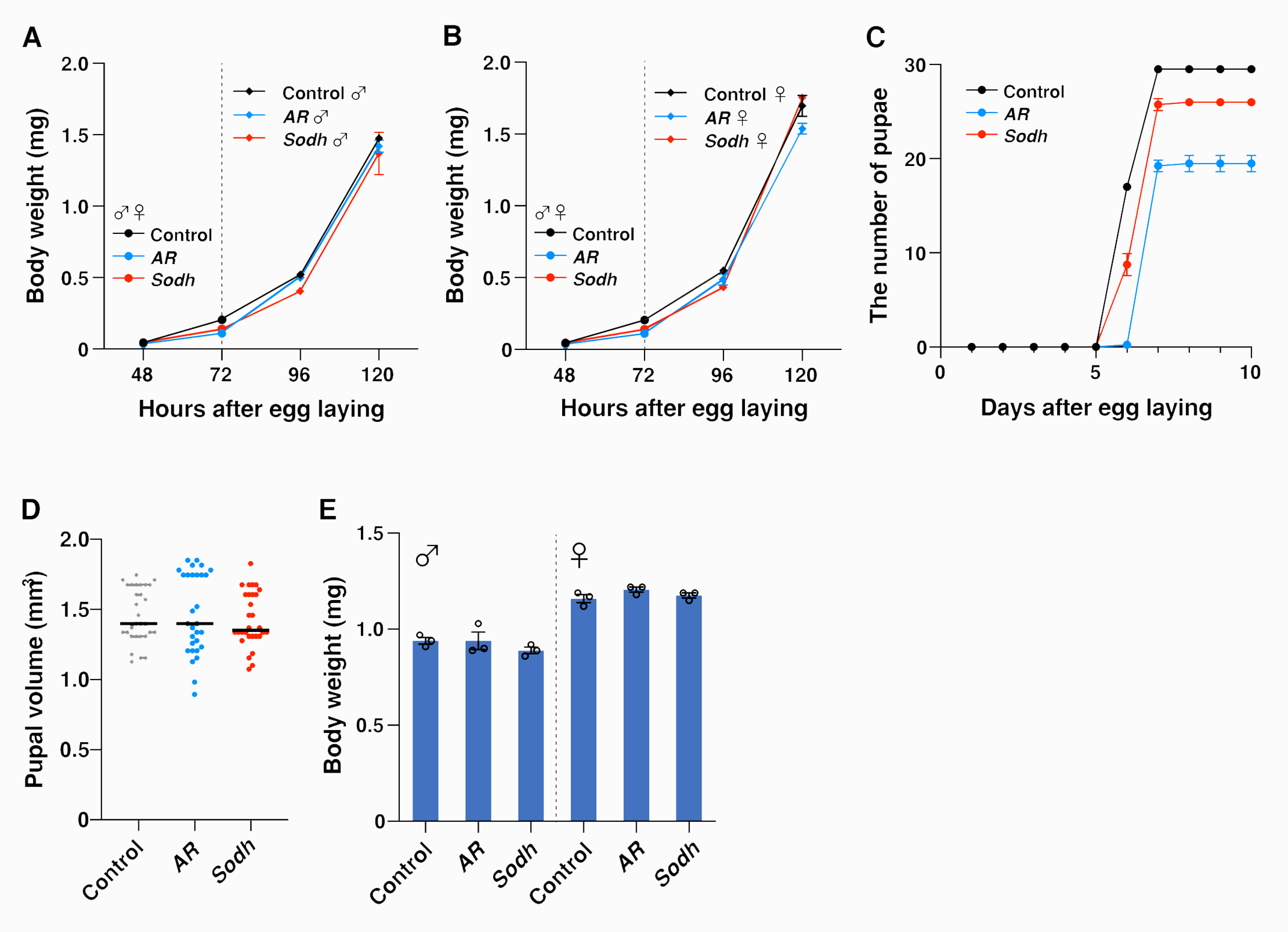

Supplement: S5 Fig — (A, B) Larval body weight of AR and Sodh mutants; 10–50 animals per batch, n = 3 batches. Line graphs show mean ± SE. (C) Timing of pupal formation in AR and Sodh mutants. Thirty larvae were grown per vial, and the number of pupae was scored every 24 hours. n = 3 vials. The line graph shows mean ± SE. (D) Pupal volume of AR and Sodh mutants. n = 30 animals. Bars in the scatter plot indicate median. (E) Adult body weight of AR and Sodh mutants. Thirty animals per batch, n = 3 batches. The bar graph shows mean ± SE. The data underlying the graphs can be found in S1 Data. AR, aldose reductase; Sodh, sorbitol dehydrogenase. (TIF) [file pbio.3001678.s005.tif]

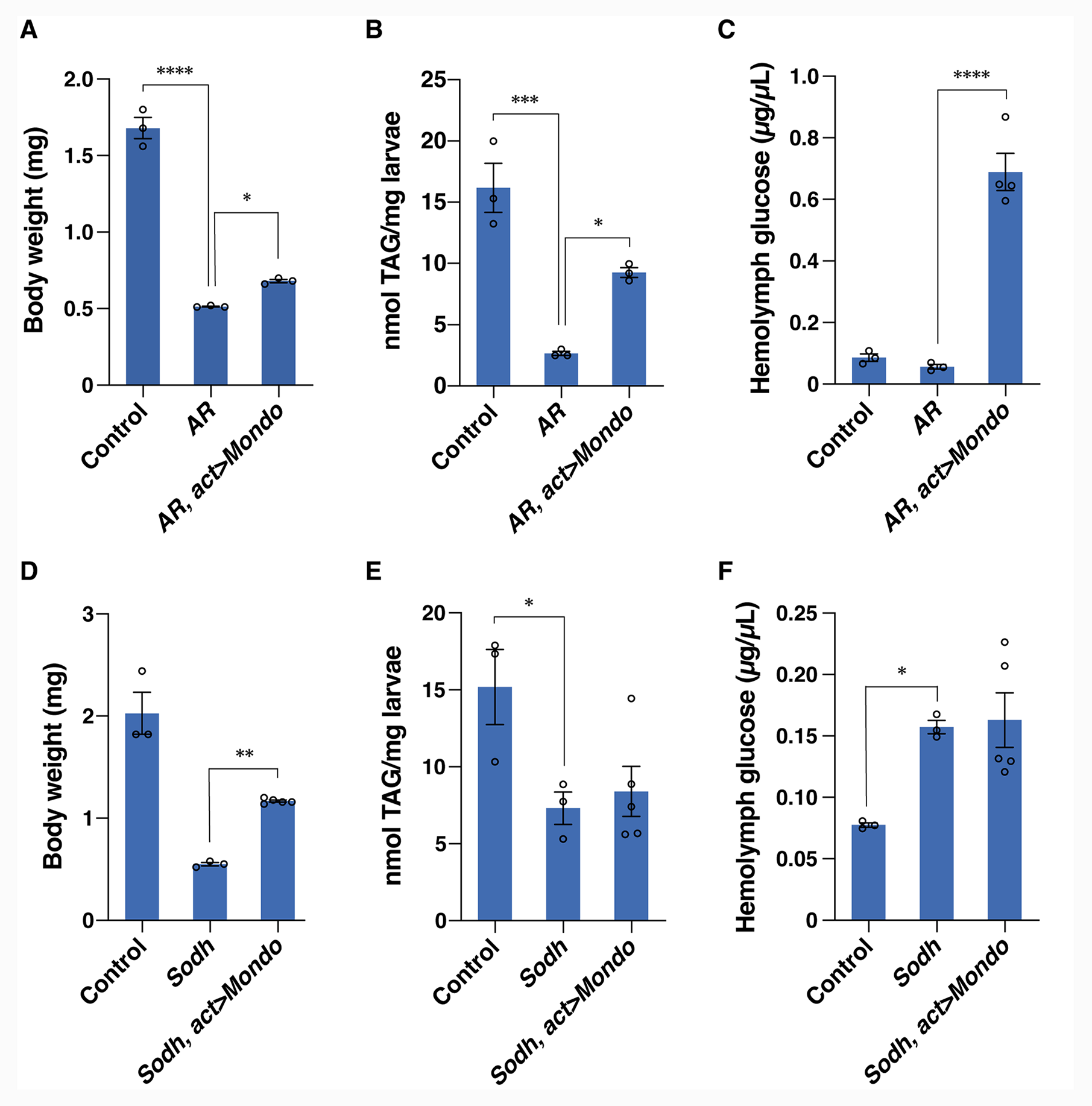

Supplement: S6 Fig — (A–C) Body weight, whole body triacylglyceride, and hemolymph glucose levels in larvae of control, AR mutant, AR mutant with ubiquitous Mondo overexpression (AR, act > Mondo). (D–F) Body weight, whole body triacylglyceride, and hemolymph glucose levels in larvae of control, Sodh mutant, and Sodh mutant with ubiquitous Mondo overexpression (Sodh, act > Mondo); 5–10 larvae per batch, n = 3 batches. Bar graphs show mean ± SE. *P < 0.05; **P < 0.01; ***P < 0.001; ****P < 0.0001. The data underlying the graphs can be found in S1 Data. AR, aldose reductase; Sodh, sorbitol dehydrogenase; TAG, triacylglyceride. (TIF) [file pbio.3001678.s006.tif]

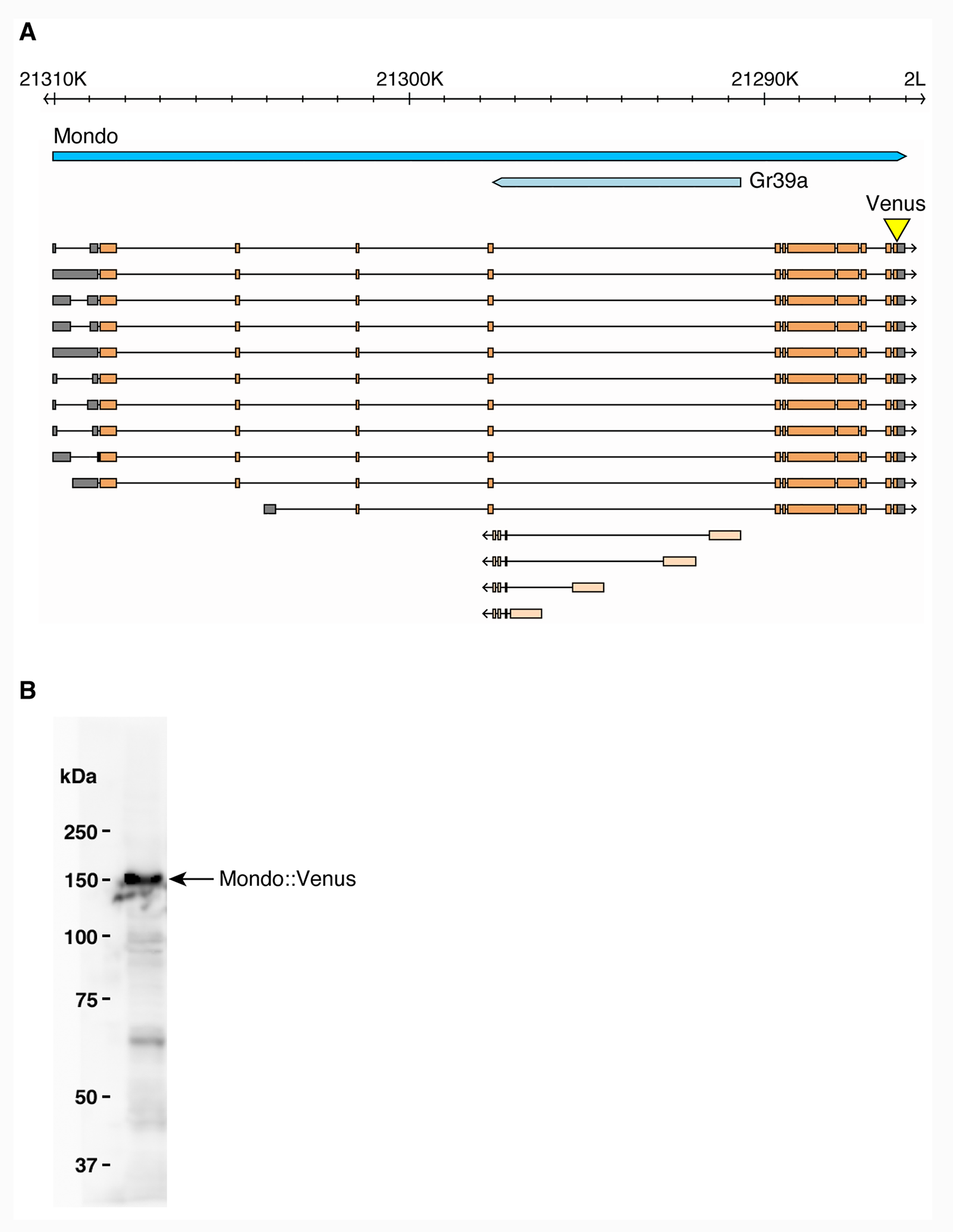

Supplement: S7 Fig — (A) Schematic drawing of the Mondo locus (adapted from FlyBase, http://flybase.org). The Venus fluorescent protein was knocked-in at the C-terminus of the Mondo coding region (yellow). (B) Western blot using fat body extracts from the Mondo::Venus line. The Mondo::Venus fusion protein was detected with the anti-GFP polyclonal antibody. Original uncropped western blot image can be found in S1 Raw image. (TIF) [file pbio.3001678.s007.tif]

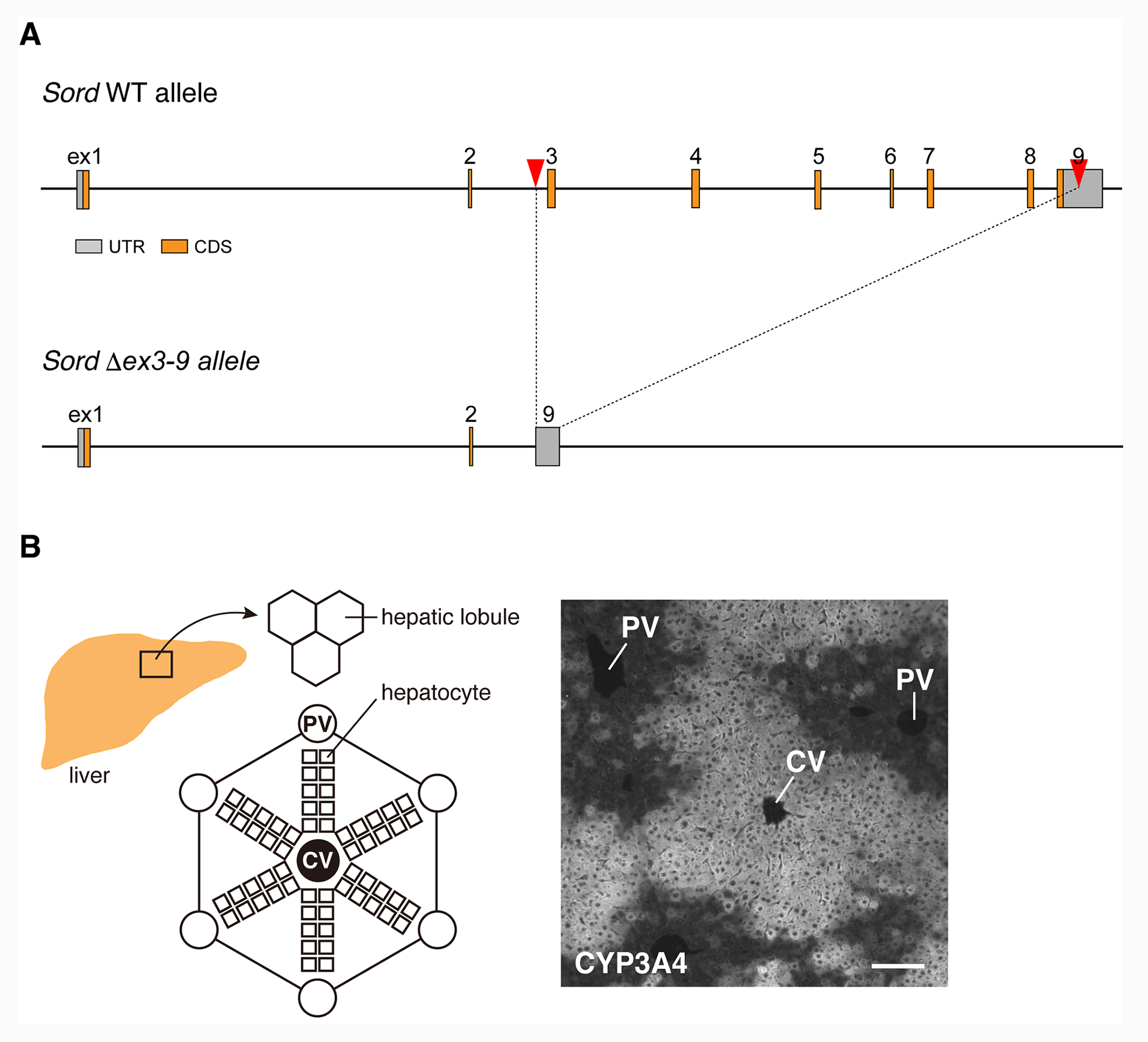

Supplement: S8 Fig — (A) CRISPR-mediated knockout of Sord. A crRNA was designed for the sequences in the intron 2 and the exon 9 of the Sord gene, resulting in the deletion from the exon 3 to the middle of the exon 9. (B) Frozen liver sections were stained with anti-CYP3A4 antibody to label pericentral hepatocytes. Regions of interest were set on the CYP3A4-positive area for quantification of ChREBP signals in pericentral hepatocytes. CV and PV are indicated in the picture. Scale bar represents 100 μm. ChREBP, carbohydrate response element-binding protein; CV, central vein; PV, portal vein. (TIF) [file pbio.3001678.s008.tif]

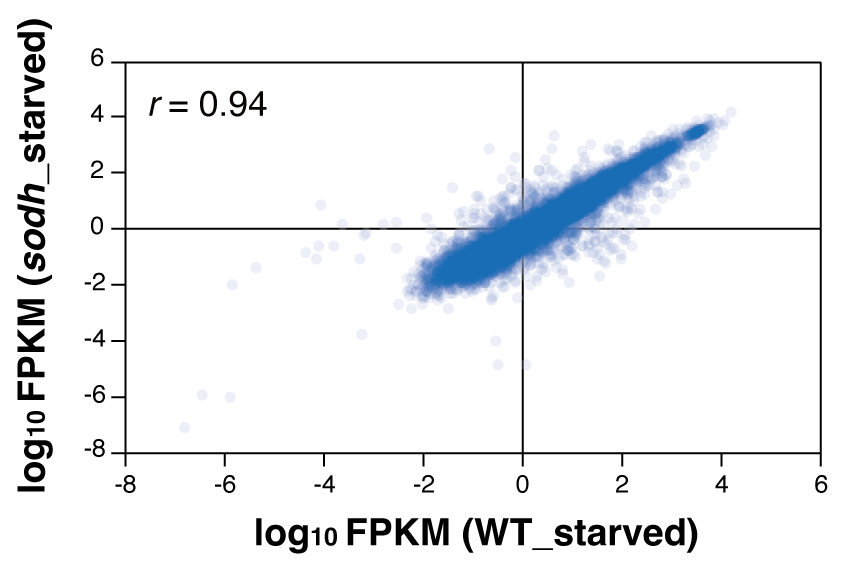

Supplement: S9 Fig — A comparison of the transcriptomes of starved wild-type and Sodh mutant larvae; 30 larvae per batch, n = 3 batches. Correlation coefficient (r) is indicated in the plot. The data underlying the graphs can be found in S9 Data. (TIF) [file pbio.3001678.s009.tif]
