# Supplementary material for: The polyol pathway is an evolutionarily conserved system for sensing glucose uptake
Source: PLoS Biol. 2022 Jun 10;20(6):e3001678. doi: 10.1371/journal.pbio.3001678 (PMC9223304; doi:10.1371/journal.pbio.3001678)
Supplement: S1 Raw image — The original uncropped western blot image of fat body extracts from the Mondo::Venus line. (PDF) [file pbio.3001678.s019.pdf]

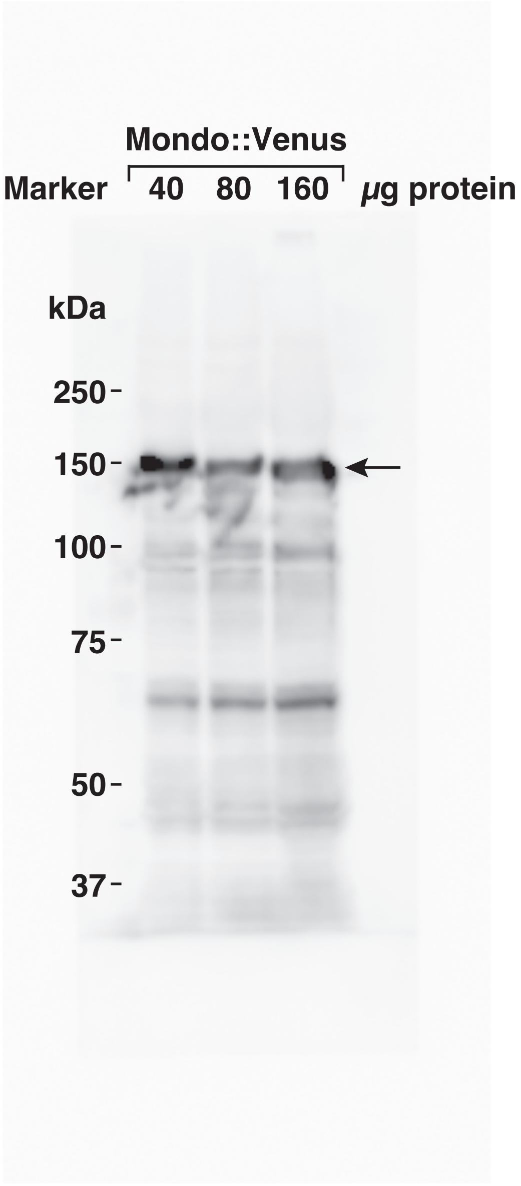

S1 Raw image. Raw image of the Western blot shown in S7B Fig.
Fat body extracts from the Mondo::Venus line were used for the Western blot.
The Mondo::Venus fusion protein was detected with the rabbit anti-GFP antibody (arrow).
