# Supplementary material for: The polyol pathway is an evolutionarily conserved system for sensing glucose uptake
Source: PLoS Biol. 2022 Jun 10;20(6):e3001678. doi: 10.1371/journal.pbio.3001678 (PMC9223304; doi:10.1371/journal.pbio.3001678)
Supplement: Attachment — Submitted filename: 120609_1_rebuttal_2243060_r3tmx1.pdf [file pbio.3001678.s020.pdf]

Revision Plan

Manuscript number: RC-2021-01065
Corresponding author(s): Hiroko Sano

1. General Statements [optional]

2. Description of the planned revisions

We greatly appreciate the reviewer's suggestions, which have helped us to improve our manuscript. Here is a point-by-point description of our plan for addressing the criticisms from the reviewers.

Reviewer #1
Major comments:
1. CCHa2 has previously been shown to be expressed in the fat body, and is a regulator of body size via its effects on the IPC cells. This study generates two mutants Sodh and AR, the authored looked at the response of these mutants to food lacking glucose, however how the lack of these enzymes contribute to body weight changes and TAG level alterations were not assessed. Do these mutants also have altered body size, Dilp expression etc? Larval growth and TAG levels were characterised, but it is not shown whether this is linked to Dilp expression, is there developmental delay, or is there a difference in growth rate?
We will examine dilp expression, growth rate, and developmental timing in *AR* and *Sodh* mutants.

2. How is body weight and TAG regulated by the polyol pathway? Is it via fat body Mondo? To show this is the case, epistasis test should be done. E.g. overexpressing or suppress Mondo target genes in AR or Sodh mutants, and assess whether the larval growth and the TAG phenotype can be rescued.
We found that the growth and TAG phenotypes in *AR* mutants were partially restored by fat body-expressed Mondo Defects in larval growth in *Sodh* mutants were also partially rescued by overexpression of Mondo in the fat body. We have shown these data in Fig. 2 of the revised manuscript. Partial rescue could be due to Mondo's function in tissues other than the fat body. To test this possibility, we are planning to perform rescue experiments using actin-Gal4/UAS-Mondo that expresses Mondo ubiquitously.

Please also see our response in pages 3 and 7.

4. Given the role CCHa2 plays in multiple tissues, it is possible that Sodh and AR mutant growth phenotype is due to changes in tissues other than the fatbody. If the authors wanted to attribute to the effects of these enzymes on overall metabolism to their function in the liver, RNAseq should be done in the fatbody. To demonstrate metabolic pathways are altered by polyol pathway, a graph showing pathway analysis would be useful.
Although we did not claim that growth and metabolic phenotype shown in Fig. 2 are attributed to the changes only in the fat body, we are interested in the point raised by the reviewer. It has been shown that Mondo functions mainly in the fat body (Havula *et al*., 2013); however, Mondo's function in other tissues are not formally excluded. The rescue experiment proposed as a response to the comment-2 will clarify whether the metabolic phenotype observed in Fig. 2 originates from changes in the fat body or from the whole body tissues.

5. The paper suggests that nuclear localisation of Mondo is functionally important. Mondo function should be link it to growth response. Genetic epistasis tests between Mondo and Sodh and AR mutant would shed light on this.
We think that the rescue experiment proposed in response to the comment-2 will answer this question.

6. Feeding is used as the main assay to look at glucose response. However, the ms does not distinguish fatbody specific effects vs overall effects in other tissues, and how multiple tissues contribute to the glucose response. This should be addressed.
We think that the rescue experiment proposed in response to the comment-2 will answer this question.

Minor comments:
1. How does starvation affect CCHa2 expression? Fig. 1B-C should include a wildtype control.
We previously showed that starvation reduces *CCHa2* mRNA levels (Sano *et al.*, PLOS Genetics, 2015). For the control in Fig. 1B, we will use cg>gfp RNAi. Wild type was used as a control in Fig. 1C.

2. Fig.1A it's better to use cg> control RNAi as controls. Otherwise, you need to demonstrate that there is no difference of the CCHa2 mRNA level between cg>+ and cg> control RNAi.
We will use cg>gfp RNAi as controls.

3. Figure S4 should be better explained in the text. Statistical analyses should be carried out.
The experiment will be performed in triplicate and include statistical analysis. Please also see our response in page 3.

Reviewer #2
Major points:
2. It is difficult to interpret the data in Fig 1 and 2 on the AR and Sodh mutants showing the effect on larval growth/physiology without an assessment of developmental rate or timing. The phenotype is only assessed at a single timepoint in the mid-third instar. The effect on body weight could be attributed to a developmental delay, for example.
We will examine growth rate and developmental timing in *AR* and *Sodh* mutants.

4. The experiment in Fig 4 is problematic because we are now comparing ex vivo culture with in vivo feeding experiments and the two have very different conditions that are not exactly comparable. The in vivo data on gene expression (Fig 1+3) are from animals starved and then fed particular sugars, whereas the ex vivo data are from fed animals given high additional concentrations of sugar in culture (overfed conditions). The better alternative would be to fix and stain fat bodies from the same in vivo conditions (starved and then fed sugars) or even an in vivo feeding of high concentrations of sugars without starving, ie hypercaloric diet. Can the authors please explain their logic, especially since the comparable experiment would be feasible and is often done (for example, Song et al Cell Metabolism 2017)? Shown as is, there is clearly a problem, because in Fig 4A+D, Mondo is in the nucleus upon addition of fructose but not glucose in a Sodh mutant. This result is hard to reconcile with the data in Fig 3F showing that fructose and glucose-treated animals show a similar gene expression response, especially the known Mondo targets. Either there are Mondo-independent effects on gene expression, even for the known Mondo targets, or the very different conditions upon which the cells were exposed to sugar affect the Mondo localization and gene expression response. Therefore, I'm not sure the authors can make the conclusion on lines 219-23 that the polyol pathway is required for activation of Mondo and that the polyol pathway acts as "a system for sensing glucose uptake that allows metabolic remodeling." This conclusion seems to extend from Fig 4 to also include Fig 3 (data on gene expression), which was done under very different conditions. It would have been useful to have the same conditions to compare in vivo and ex vivo.
We agree that data on gene expression and Mondo's nuclear localization should be compared under the same conditions. Thus, we are planning to examine sugar-dependent nuclear localization of Mondo using *ex vivo* culture of fat bodies dissected from starved larvae.

Minor points:
4. In Fig S4A, consider plotting the sorbitol data in a different way, for example with a break in the y-axis - right now it is difficult to compare w, AR, and OR on this axis choice. Same can be said for part B.
We will perform GC/MS in triplicate according to Reviewer#1's minor commnent-3. The data will be displayed as suggested.

Reviewer #3
Major comments:
Given that the authors investigate glucose sensing by Mondo/ChREBP and the involvement of the polyol pathway, experiments determining how much of the glucose pool is metabolized through the polyol pathway (in the biological systems and under the basal conditions used by the authors) are missing. Since the experiments involving sorbitol

are only with exogenous sorbitol, it is difficult to know whether intracellular endogenous sorbitol, which is derived from glucose, exerts the same effect (is intracellular endogenous sorbitol sufficiently concentrated as compared to exogenous sorbitol?). Flux analysis using labeled glucose to trace and measure the proportion of glucose molecules entering the polyol pathway to be converted to sorbitol will be extremely informative in that regard. Also, quantifying the amount of intracellular endogenous sorbitol in glucose starved versus basal conditions will be useful.
The authors examined the effect of xylitol on the induction of CCHa2 expression, it will be informative if they could check at the impact of xylulose.
We have shown that glucose-dependent nuclear translocation of Mondo is inhibited in AR mutants in which the conversion of glucose to sorbitol is disrupted (Fig. 4A and C in the original manuscript). These data provide evidence for the involvement of endogenous sorbitol in Mondo activation. Nonetheless, flux analysis suggested by the reviewer #3 will be informative to confirms that sorbitol is produced from ingested glucose and the proportion of glucose that enters the polyol pathway. Therefore, we will perform flux analysis using ^{13}C-labeled glucose.

3. Description of the revisions that have already been incorporated in the transferred manuscript

We have made corrections in the text to accommodate the comments made by the reviewers. We have also revised Fig. 2 and 6.

Reviewer #1

Major comments:

2. How is body weight and TAG regulated by the polyol pathway? Is it via fat body Mondo? To show this is the case, epistasis test should be done. E.g. overexpressing or suppress Mondo target genes in AR or Sodh mutants, and assess whether the larval growth and the TAG phenotype can be rescued.
We found that the growth and TAG phenotypes in *AR* mutants were partially restored by fat body-expressed Mondo Defects in larval growth in *Sodh* mutants were also partially rescued by overexpression of Mondo in the fat body. We have shown these data in Fig. 2 of the revised manuscript.

3. In the mutant, the activation of CCHa2 is altered, but to prove definitively that AR metabolises glucose to give rise to sorbitol and sodh metabolises sorbitol to fructose, the metabolic products should be quantified in the mutant fatbody or in circulation.
We have quantified levels of glucose, sorbitol, and fructose in the hemolymph of *AR* and *Sodh* mutant larvae. The data has been shown in Fig. S4 in the original manuscript.

Minor comments:

3. Figure S4 should be better explained in the text. Statistical analyses should be carried out.
We have added explanation of the GC/MS data in the revised manuscript (lines 150-151).

4. Line 127: "but also its induction upon glucose ingestion (Fig. 1B). This is glucose refeeding after 18-hour starvation.
We have rephrased the sentence to “It also reduced *CCHa2* expression upon glucose refeeding after 18-hour starvation, indicating that Mondo is required for acute induction of *CCHa2* expression in response to glucose ingestion (lines 125-127 in the revised manuscript).”

5. Line127 "but also its induction upon glucose ingestion" what does this suggest? Fig.1B needs clarification.
We have rephrased the sentence to “It also reduced *CCHa2* expression upon glucose refeeding after 18-hour starvation, indicating that Mondo is required for acute induction of *CCHa2* expression in response to glucose ingestion (lines 125-127 in the revised manuscript).”

6. Line 151: "Even though genes involved in glycolysis and PPP were intact in these animals", quantifications should be carried out.

In this sentence, we meant that genes involved in glycolysis and PPP are not mutated in mutant of AR or Sodh. We deleted this sentence as it may mislead readers into thinking that the activity of enzymes involved in glycolysis and PPP was intact.

7. Line 162-163: according to Fig2.B, it should be loss of whole larval triglycerides.
We have changed “abnormal triacylglycerol accumulation” to “loss of whole larval triglycerides (line 153 in the revised manuscript).”

8. Line 174-175: what are polyol pathway metabolites?
We have changed “polyol pathway metabolites” to “metabolites generated by the polyol pathway (lines 166-167 in the revised manuscript).”

9. Line 190 and 193: "Fig 2F" should be "Fig. 3F
We have corrected the figure number.

10. Fig. 4A. It would help to have arrows pointing to the nucleus. It would also be good to show that Mondo nuclear intensity is decreased in AR and Sodh mutants. I understand that this change could not obviously be seen here because images in top, middle and bottom panels are not comparable. But I would recommend including a comparison of Mondo nuclear localization of control, AR mutant and Sodh mutant and provide some quantifications.
We have added arrowheads in Fig. 4A to indicate the nucleus.
Please also see our response in page 7.

11. A summary schematic should be included to better explain the findings.
In Fig. 6, we have added a schematic diagram showing the function of the polyol pathway.

Reviewer #2
Major points:
1. The first claim - that polyol pathway is required for Mondo-mediated CCHa2 expression - is actually only true for the fed state, not the starved. The best data to support this claim are in Fig 1F, showing that CCHa2 mRNA levels are reduced in AR and Sodh mutants. However they show that glucose is sufficient for the response upon starvation. The authors admit this limitation in lines 155-158. However, concluding statements in the manuscript seem to neglect this important point and imply that the polyol pathway is always required, which is misleading, specifically, the title of the first results section, first figure and lines 103-4 at the end of the introduction. These statements should be amended.
To solve the problem, we moved Fig. 1D and 1F after Fig. 3F. In Fig. 1, we claim that Sorbitol feeding induces Mondo-mediated *CCHa2* expression. The title of the first result section and Fig.1 was changed accordingly.
We have also rephrased the sentence in lines 103-104 in the original manuscript to “the polyol pathway is involved in the activation of Mondo/ChREBP (lines 103 in the revised manuscript).”

3. Related to point 1, it is not clear how the authors support the conclusion drawn on lines 182-4. Fig 3E-F indicates that glucose alone can rescue much of the transcriptional response of the Sodh mutant, in particular the response of the genes highlighted as known Mondo-targets in 3F. Thus, the authors cannot claim that the polyol pathway alone can play "an essential role" in Mondo-mediated transcriptional regulation. Something similar also appears in lines 112-3 at the end of the introduction and lines 193-4. The authors themselves admit on lines 198-9 that there are glucose-metabolizing pathways other than the polyol pathway that can activate Mondo under starved conditions. Thus, they are aware of this caveat, but nonetheless they consider the polyol pathway "essential". The data in Fig 1F do suggest that under fed conditions the polyol is important under fed conditions, but the bulk of their data, including the sequencing shown in Fig 3 comes from the starved state, where the polyol pathway does not seem to be essential.
We agree with the reviewer’s suggestion and have rephrased the texts as follows.
Lines 112-113 in the original manuscript: The polyol pathway <u>regulates</u> Mondo-target metabolic genes (line 109 in the revised manuscript).

Lines 182-184 in the original manuscript: These results show that the polyol pathway can mediate sugar-dependent expression of Mondo/Mlx-target genes (lines 174-175 in the revised manuscript).
Lines 193-194 in the original manuscript: These results show that the polyol pathway can induce the expression of various metabolic enzymes (lines 184-185 in the revised manuscript).

As we mentioned in major comment-1, we moved Fig. 1D and 1F after Fig. 3F, and have added explanation of differential requirements of the polyol pathway in different nutritional conditions (lines 186-194 in the revised manuscript).

5. The discussion is somewhat difficult to read and wanders a bit too far from the work shown in this paper. It could benefit from some editing to make it more focused on the exact findings and limitations of this study. For example,
• the logic of the first paragraph is unclear.
We have revised the first paragraph in Discussion (lines 238-245 in the revised manuscript).

• In lines 254-46, the statement does not seem supported by this study - there is no data in this paper showing precisely that the activity of the metabolic pathway required for sugar sensing is correlating with the levels of glucose in the body fluid.
We did not claim that we showed that the activity of the metabolic pathway(s) required for sugar sensing correlates with the levels of glucose in the body fluid. We meant that such a theory has been generally accepted.

• In lines 256-8, what data or reference justifies saying that the polyol pathway is the "most upstream glucose-metabolizing pathway"? The authors themselves state in line 248 that there is a low affinity of AR for glucose (although it is not clear what "low" is in comparison to). If the authors choose to include this statement, then they need to explain the logic of how that allows the polyol pathway to be an efficient glucose-sensing mechanism.
The statement of “the most upstream glucose metabolizing pathway” was incorrect. We have changed it to “the polyol pathway metabolizes glucose immediately after it enters the cell (lines 250-251 in the revised manuscript).”
“Low affinity” means that affinity of AR for glucose is much lower than that of hexokinase for glucose. We have corrected the statement (lines 238-239 in the revised manuscript).

• Lines 279-89: it is not exactly clear what point the authors are trying to make and why this section is relevant to this work.
We have revised this paragraph focusing on the benefit of using fructose to detect glucose uptake (lines 278-292 in the revised manuscript).

• The entire section of lines 302-342 (Insights into fructose-induced pathogenic mechanisms) reaches quite far from the work shown here, especially as it tries to directly relate the data shown here to other observations of human physiology. For example, the statement on lines 307-9 is a stretch. The "metabolic remodeling" shown here comes mostly from the transcriptional data in Fig 3 on starvation, and the only data in mice shown here is the ChREBP localization upon sugar feeding after starvation. Saying that these results account for the phenomenon of fructose improving glucose tolerance in healthy and diabetic adults (human? Or mice? Also not clear) is not reasonable. I strongly advise that this section be either deleted, or at least considerably shortened.
We agree that lines 302-342 in the original manuscript reaches far from what we have shown. Therefore, we have substantially revised and shortened this paragraph (lines 294-310 in the revised manuscript).

Minor points:
1. Line 119: "what might activates" is grammatically incorrect - subject/verb agreement.
We have corrected it.

2. Fig1: reference to Histograms, but these are not histograms, rather bar graphs.
We have corrected it.

3. Consider putting the data in Fig 1F before the starvation data.

We have moved Fig. 1F after the transcriptome data in Fig. 3 to avoid confusion about the experimental conditions.

5. Lines 190 and 193: wrong figure reference - should be Fig 3F, not 2F (which doesn't exist).
We have corrected the figure number.

6. The quantification done for Fig 4 should be explained in more depth - simply stating what software was used is not sufficient. Rather it should be reported what exactly was done (eg., were the regions highlighted by hand and then intensity quantified? Was there rather a script for automatic segmentation?, etc).
We have added detailed method regarding the quantification done for Fig. 4 as described below .
"Images of the fat body were analyzed using the ImageJ2 software (version 2.0.0-rc-43, NIH). We traced the contours of the cell and nucleus with the freehand tool and measured the intensity of the signal contained within each region, then the percentage of nuclear Mondo was calculated (lines 512-515 in the revised manuscript)."

7. Typo in Fig 5O ("contol")
We have corrected it.

8. There seem to be quite a few instances in the discussion where a reference should be included to support the claim, e.g., lines 246-7, 257-9, 284-5
Lines 246-247 in the original manuscript: We have cited the following references (lines 244-245 in the revised manuscript).
Kanehisa, M. and Goto, S. KEGG: kyoto encyclopedia of genes and genomes. Nucleic Acid Res 28, 27-30 (2000).
Kanehisa, M. Toward understanding the origin and evolution of cellular organisms. Protein Sci 28, 1947-1951 (2019).
Kanehisa et al. KEGG: integrating viruses and cellular organisms. Nucleic Acids Res. 49, D545-D551 (2021).

Lines 257-259 in the original manuscript: The statement of "The polyol pathway is the <u>most upstream</u> glucose-metabolizing pathway." was incorrect as pointed by the reviewer in major comment-5. Thus we have changed as follows: The polyol pathway metabolizes glucose immediately after it enters the cell, and the above references for KEGG have been cited (lines 250-252 in the revised manuscript).

Lines 284-285 in the original manuscript were deleted.

Reviewer #3
<u>Major comments:</u>
The experiments presented in this manuscript are properly designed, technically sound and the cellular and animals models used are appropriate. Overall, most but not all the conclusions of the manuscript are convincing. The authors provide clear evidence that sorbitol and fructose, metabolites of the polyol pathway, are inducing Mondo activity. Regarding the claim that glucose activates Mondo and ChREBP through its metabolization by the polyol pathway, some of the data are seemingly contradictory. On one hand, a number of experiments are supportive ("The authors found that in Drosophila glucose induced nuclear translocation of Mondo is prevented by mutations of Sodh or of aldehyde reductase (AR) (Figure 4), another polyol pathway enzyme encoding gene. This was further demonstrated in mouse models, as glucose induced nuclear translocation of ChREBP in hepatocytes was only observed in wild type animals, but not in sorbitol dehydrogenase Sord knock out animals (Figure 5)."). On the other hand, some other data are arguing against this claim ("glucose-mediated activation of Mondo target genes (Figure 3), including the typical CCHamide-2 CCHa2 target (Figure 1D), was not significantly reduced in Sodh mutants as compared to wild type flies"). Therefore, the author should clearly specify that glucose metabolization by the polyol pathway induces nuclear translocation of Mondo and ChREBP but not Mondo transcriptional activity and provide potential explanations to explain this point.
We agree that we should clarify what the polyol pathway is required for and under what conditions. Thus, we moved Fig. 1D and 1F after Fig. 3F, and have added explanation of differential requirements of the polyol pathway in different nutritional conditions (lines 186-194 in the revised manuscript).

The authors mentioned in the introduction that "Most of glucose-induced nutritional adaptation is the results of glucose-dependent transcription". This is disregarding the importance of post-translational (signaling pathways and

enzymatic activities) and translational reprogramming induced by glucose. The authors should balance such a claim in their introduction to reflect the broader range of biological responses induced by glucose.
We have rephrased the sentence to "Glucose-dependent transcription is an important mechanisms of glucose-induced nutritional adaptation (lines 62-63 in the revised manuscript)."

Minor comments:
- Figure 1D is described after Figures 1E and 1F in the results section
We have arranged the figures according to the order in which they appear in the text.

- Figure 3F is improperly labelled Figure 2F in the results section (page 7, lines 190 and 193)
We have corrected the figure number.

4. Description of analyses that authors prefer not to carry out

Reviewer #1
Major comments:
2. How is body weight and TAG regulated by the polyol pathway? Is it via fat body Mondo? To show this is the case, epistasis test should be done. E.g. overexpressing or supress Mondo target genes in AR or Sodh mutants, and assess whether the larval growth and the TAG phenotype can be rescued.
As Reviewer #2 pointed out, rescue experiments by over-expressing or suppressing a small set of Mondo target genes would be unsuccessful, as there are numerous Mondo target genes (Fig. 3). Therefore, we would like to decline to do this experiment.

Minor comments:
10. Fig. 4A. It would help to have arrows pointing to the nucleus. It would also be good to show that Mondo nuclear intensity is decreased in AR and Sodh mutants. I understand that this change could not obviously be seen here because images in top, middle and bottom panels are not comparable. But I would recommend including a comparison of Mondo nuclear localization of control, AR mutant and Sodh mutant and provide some quantifications.
The purpose of Fig. 4 is to compare the nuclear localization of Mondo in wild type and mutants of AR, Sodh. We think the percentage of nuclear Mondo is the best indicator. Because the expression level of Mondo may differ among wild type, AR, and Sodh mutants, and staining variations between different samples may occur, comparing the amount of Mondo in the nucleus would be unsuitable for the purpose.

Reviewer #3
Major comments:
The authors examined the effect of xylitol on the induction of CCHa2 expression, it will be informative if they could check at the impact of xylulose.
We have shown that xylitol ingestion does not induce CCHa2 expression, strongly suggesting that xylitol metabolism is not involved CCHa2 expression (Fig. S4C). Therefore, we think there is little point to test the effect of xylulose.
