# Supplementary material for: The polyol pathway is an evolutionarily conserved system for sensing glucose uptake
Source: PLoS Biol. 2022 Jun 10;20(6):e3001678. doi: 10.1371/journal.pbio.3001678 (PMC9223304; doi:10.1371/journal.pbio.3001678)
Supplement: Attachment — Submitted filename: Response to Reviewers_3.docx [file pbio.3001678.s023.docx]

**Response to Reviewers**

We are grateful to the Reviewer #2 for their helpful suggestions. Below is a point-by-point response to the criticisms.

1. When describing the phenotype on larval growth of the AR and Sodh mutants, the authors describe "weight loss" on several occasions, but actually it's just a decline in growth rate (there is not a loss or decline in the actual weight).

We agree with the reviewer’s suggestion and have rephrased the texts as follows.

Lines 162-163 in the original manuscript: *AR* and *Sodh* mutant larvae displayed a slight decrease in growth rate (lines 163-164 in the revised manuscript).

Line 167 in the original manuscript: The mutant larvae showed a marked reduction in growth rate during larval stages (lines 168-169 in the revised manuscript).

2. The authors have mostly addressed my previous comment that the polyol pathway is not absolutely required (depends on fed/starved state) -- but there is still rather strong language in the abstract: lines 42-3 "depends on", 45-6 "is required". Consider softening/qualifying these statements.

Lines 42-43 in the original manuscript: ChREBP/MondoA activation in response to glucose ingestion involves an evolutionarily conserved glucose-metabolizing pathway: the polyol pathway (lines 42-43 in the revised manuscript).

Lines 45-46 in the original manuscript: We show that the polyol pathway regulates the glucose-responsive nuclear translocation of Mondo (lines 45-46 in the revised manuscript).

3. There is still something I don't understand in the discussion: you mention that the affinity of AR for glucose is very low compared to that of hexokinase (339-40), but then you say that "glucose flows into the polyol pathway before being metabolized to G6P..." (no reference for this statement by the way). Can you please explain how that works for me -- and/or maybe cite a reference?

The statement of “Glucose flows into the polyol pathway before being metabolized to glucose-6-phosphate, which is consumed through glycolysis and PPP (lines 351-353 in the original manuscript)” was deleted as it is inaccurate.
